# Non-Coding RNAs in Primary Liver Cancer

**DOI:** 10.3389/fmed.2015.00036

**Published:** 2015-06-04

**Authors:** Michele Ghidini, Chiara Braconi

**Affiliations:** ^1^Division of Cancer Therapeutics, The Institute of Cancer Research, London, UK; ^2^Cancer Center, Humanitas Clinical and Research Center, Milano, Italy; ^3^The Royal Marsden NHS Foundation Trust, London, UK

**Keywords:** non-coding RNA, microRNA, long non-coding RNA, HCC, liver cancer

## Abstract

Hepatocellular carcinoma (HCC) is a primary malignancy of the liver with poor prognosis and limited therapeutic options. Over the past few years, many studies have evaluated the role of non-coding RNAs (ncRNAs) in hepatocarcinogenesis and tumor progression. ncRNAs were shown to have diagnostic, prognostic, and therapeutic potential in HCC. In this manuscript, we review the latest major discoveries concerning microRNAs and long ncRNAs in HCC pathogenesis, and discuss the potentials and the limitations for their use in clinical practice.

## Hepatocellular Carcinoma

Hepatocellular carcinoma (HCC) is a primary tumor of the liver and represents the third cause of cancer deaths worldwide (1). Only few patients are eligible for curative treatments, while majority of cases are diagnosed at later stages (1). HCCs often arise on a background of liver cirrhosis and, as such, early diagnosis is frequently missed. Local ablative therapies, such as transarterial chemoembolization (TACE) and radiofrequency ablation (RFA), are used when the tumor is localized within the liver, while the multikinase-inhibitor sorafenib is the only approved systemic therapy for advanced HCC (2, 3). However, overall survival (OS) of patients affected by HCC remains poor. A growing effort has been addressed toward the study of genomics and molecular biology in order to unravel the mechanisms of liver carcinogenesis and therefore identifying novel targets of therapies as well as early diagnostic and prognostic markers to improve the clinical management of HCC patients. Along with an extensive characterization of the protein-coding genome of liver tumors (4–7), there has been a great interest in the study of non-coding RNAs (ncRNA). Hereby, we will review the role of ncRNA in liver carcinogenesis and their clinical implication.

## Non-Coding RNAs in HCC

Most of the eukaryotic genome is transcribed into RNA transcripts that do not translate into proteins. These RNA transcripts can be generally divided into two classes according to their size (with 200 nt as cut-off): short ncRNAs, including microRNAs (miRNAs) and long non-coding RNAs (lncRNAs). Although they are not translated into protein products, they exert essential functions within the cell by modulating the expression of protein-coding mRNAs, interacting with proteins to affect their function, and interacting one with each other to finely tune their expression (8).

### microRNAs

microRNAs are 18–24 nt long and represent key actors in the processes of tumor development, progression, and resistance to anti-tumor agents (9). Many studies have identified alterations in miRNAs in HCC (10–12). Some miRNAs, such as miR-21 and miR-221/222, were found increased in expression, and acting as oncogenes by affecting several cancer-related pathways (13–18). On the other hand, selected miRNAs, such as miR-26, miR-29, miR-122, miR-148a, and miR-199a, were reported to be reduced in HCC and to promote cancer by the lack of their oncosuppressive activity (19–24).

Data on the expression of miRNAs have mainly come from real time PCR-based and microarray-based profiling. More recently, the introduction of novel technologies, such as RNA next generation sequence (NGS) (RNA-seq), has led to the identification of novel miRNAs. NGS analysis produces miRNA expression profile that is reproducible and comparable to that produced by microarray, but has the advantage of discovering novel miRNAs (as for the case of miR-9986 for HCC), and of providing a detailed profile of expression of miRNA isoforms (25, 26). If down-regulation of miR-199a-3p in HCC was a solid data achieved through old technologies (27, 28), Wojcicka et al. showed through NGS that there are nine different isoforms of miR-199a-3p, which include three different seed regions. All the miR-199a-3p isoforms are lower in HCC tumors, and apparently the gene targeting is conserved across the isoforms (26). These findings add interesting insights into the understanding of miRNAs, because they imply that the message delivered by each miRNA may be amplified according to the expression of their isoforms, and probe designing should take into account the variability among these.

Several reports have confirmed that the aberrant expression of miRNAs in HCC cells is associated with the derangement of a number of pathways and processes, which all together initiate and maintain cancer, promote cell growth, mediate apoptosis escape, and induce migration and invasion (Table 1). An example among all is miR-21, whose over-expression results into the silencing of several targets including phosphatase and tensin homolog (PTEN), programed cell death protein 4 (PDCD4), reversion-inducing-cysteine-rich protein with kazal motifs (RECK), metalloproteinase inhibitor 3 (TIMP-3), and Pellino-1 (13, 14, 29–31), but is concomitantly finely tuned by a plethora of factors whose source can either be the tumor cell itself [nuclear factor kinase B (NF-KB), hepatitis B virus X protein (HBV x)] (32, 33), or microenvironment cells [interleukin-6 (IL-6) or monocyte chemotactic protein-1 (MCP-1)] (33, 34). It is interesting to note that normal liver tissue seems to express a limited number of miRNAs, including miR-199a and miR-122 (35). Thus, it is not surprising that HCC tissues exhibit loss of these miRNAs along with over-expression of others, such as miR-21, miR-221/222, and miR-517 (13, 18, 36).

**Table 1:** **microRNAs involved in HCC pathogenesis and development**.

microRNA	Expression in HCC compared to normal liver	Target	Cell phenotype	Source	Reference
miR-21	Up	PTEN, PDCD4, RECK, TIMP-3, Pellino-1	Growth, proliferation, invasion, and migration	Human tissues and cell lines	(13, 14, 29–31, 34)
				Mouse tissues and cell lines	
miR-155	Up	STAT-3, C/EBPbeta, SOX6, APC	Growth, progression, and invasion	Human tissues and cell lines	(138–142)
				Mouse tissues and cell lines	
miR-221/222	Up	CDKN1C/p57, MET, Bmf, PI3K-AKT-mTOR, PPP2R2A, DDIT4, *p27*, *p57*, ARNT	Growth, cell-cycle progression, proliferation, invasion, and survival	Human tissues and cell lines	(15–18, 70, 143–146)
				Mouse tissues	
miR-517	Up	NF-kB, MAPK/ERK	Proliferation, migration, and invasion	Human tissues	(36)
miR-331-3p	Up	PHLPP	Proliferation, migration, and invasion	Mouse tissues	(68)
miR-664, miR-485, miR-495	Up	MAT1A	Growth, migration, and invasion	Human cell lines	(147)
				Mouse tissues	
miR-193b	Down in HCC, Up in HCV-HCC cells	Mcl-1, Cyclin D1, ETS1	Apoptosis, invasion, migration, cell-cycle progression response to sorafenib	Human tissues and cell lines	(79, 80, 148)
				Mouse tissues	
miR-199a-3p	Down	*MET*, ERK2, CD44	Motility, invasion, and survival	Human tissues and cell lines	(27, 28, 93)
				Mouse tissues and cell lines	
miR-26a	Down	Bcl-2, Mcl-1, Cyclins D1/E2/D2, CDK6, MMP2, *MET*, VEGFA, VEGFR-2, LOXL-2, IL6, TAK1, TAB3	Proliferation, migration, cell-cycle progression, invasion, motility, and survival	Human tissues and cell lines	(19, 92, 149–151)
				Mouse tissues	
miR-29	Down	DNMT3A, LOXL-2, *MEG3*, Bcl-2, Mcl-1, SIRT1	Cell growth, survival, and proliferation	Human tissues and cell lines	(20, 41, 69, 104)
				Mouse tissues	
miR-148a	Down	DNMT1, *MET*, Myc, Snail, ACVR1/BMPs	Growth, cell-cycle progression, invasion, and migration	Human tissues and cell lines	(24, 40, 152–154)
				Mouse tissues and cell lines	
miR-122	Down	Cyclin G1, ADAM10, SRF, IGFR1, Myc, CUTL1	Growth, cell-cycle, progression, proliferation, migration, invasion, and survival	Human tissues, cell lines and sera	(21, 38, 57, 155–160)
				Mouse tissues and cell lines	
let-7 family	Down	Bcl-x, Type 1 collagen α, Myc, p16^INK4A^	Proliferation, migration, and invasion	Human tissues and cell lines	(161, 162)

*ACVR1/BMPs, activin A receptor, type 1/bone morphogenetic proteins; ADAM10, a disintegrin and metalloproteinase domain-containing protein 10; APC, adenomatous polyposis coli; ARNT, Aryl hydrocarbon receptor nuclear translocator; Bcl-2, B-cell lymphoma 2; C/EBPbeta, CCAT/enhancer binding protein beta; CDKN1C, cyclin-dependent kinase inhibitor 1C; DDIT4, DNA-damage inducible transcript 4; ERK, extracellular signal-regulated kinases; ETS1, v-ets avian erythroblastosis virus E26 oncogene homolog 1; IGFR-1, insulin growth factor receptor-1; LOXL2, lysil oxidase-like 2; MAPK, mitogen-activated protein kinase; MAT1A, methionine adenosyltransferase I, alpha; PHLPP, PH domain and leucine rich repeat protein phospathases; PPP2R2A, protein phosphatase 2, regulatory subunit B, alpha; SIRT-1, sirtuin-1; SRF, serum response factor; STAT-3, signal transducer and activator of transcription-3; TAB3, TGF-beta-activated kinase 1/MAP3K7 binding protein 3; TAK1, transforming growth factor beta-activated kinase 1; VEGFA, vascular endothelial growth factor A; VEGFR-2, vascular endothelial growth factor receptor-2*.

The mechanisms through which miRNA expression is modulated during liver carcinogenesis are variable, and include chromosomal rearrangements, promoter methylation, and transcriptional induction through direct control of transcription factors such as Myc (9, 37–40). Recent evidence suggests that alpha-feto-protein (AFP) can alter miR-29 expression and can induce changes in the methylome of liver cancer cells that are responsible for the more aggressive behavior of AFP + HCC (41). Growing evidence show that nanovesicles-mediated delivery of miRNAs is another way of miRNA regulation and intercellular communication. Tumor cells were shown to actively secrete miRNAs through exosomes, which can then be internalized by other cells altering miRNA profiling, and modulating gene expression in donor cells (42, 43). Vacuolar protein sorting-associated protein 4A (Vps4A) has been identified as one of the regulators of exosome bioactivity, as it facilitates the release of exosome containing onco-miRs and alters the accumulation of those containing oncosuppressor miRNAs (44). miRNAs are highly conserved genes, and therefore, alterations of their sequence have been studied as potential causes of aberrant miRNA expression. It was observed that selected polymorphisms in miRNA genes might result in increased production of mature miRNA forms and therefore induce miRNA-dependent liver carcinogenesis. For instance, a G > C polymorphism located in the stem region of miR-146a is associated with increased predisposition to develop HCC (45). To date, polymorphisms in a number of miRNAs have been associated to increased risk of HCC (46–51). However, these studies have been carried out mainly in Chinese or Turkish populations, and their extrapolation to other ethnicities is not clear.

#### Clinical Implications of miRNAs in HCC

##### Diagnostic role

As previously discussed early diagnosis is one of the main challenges in the clinical management of HCC. miRNAs have shown to distinguish HCC from adjacent normal or cirrhotic tissues (18, 22, 36, 52). However, the main advances have been achieved through the detection of circulating miRNAs. Tissutal analyses are indeed a limitation in this field given HCC diagnosis is made on a combination of blood tests and imaging with no recommendation for mandatory biopsy in most of cases (1). Conversely, miRNAs can be detected in sera and plasma (53). They can circulate as free RNAs, which are bound to Argonaut RISC catalytic component 2 (Ago2) or included in exosomes; in either case, they are protected by RNases and remain stable after harsh conditions (54, 55). There are now a number of reports that showed miRNAs are detectable in the plasma and sera of HCC patients and their expression profile is different between patients with HCC and patients with cirrhosis, as reviewed by Roderburg et al. (56). The larger study includes a series of 934 participants among healthy controls, and patients with chronic hepatitis B, cirrhosis, and hepatitis B virus (HBV)-related HCC (57). A panel of seven plasma miRNAs (miR-122, miR-192, miR-21, miR-223, miR-26a, miR-27a, and miR-801) was shown to have a high-diagnostic accuracy of HCC. The diagnostic performance persisted regardless of disease stage and was able to differentiate HCC from healthy controls, chronic hepatitis B, and cirrhosis (57), suggesting a potential use of this panel in the early diagnosis of HBV-related HCC. However, it looks like circulating miRNA profiles differ according to the etiology, and therefore, these data cannot be extrapolated to all cases of HCC. Indeed, circulating miRNAs not only can distinguish patients who have developed HCC among all the HBV carriers but can also distinguish between patients with HBV and those with hepatitis C virus (HCV) (58), suggesting that specific miRNAs may be identified in each subtype of HCC. These data are not surprising given that (1) miRNA expression profiles were shown to be different in tissues from HBV-related versus HCV-related HCC (59), (2) miRNAs can facilitate replication of hepatitis viruses (60, 61), and (3) circulating miRNAs can reflect the status of liver injury in inflammatory diseases (62). Nonetheless, we need to remember that circulating miRNAs may also originate from blood cells, and therefore, an appropriate sample collection and processing, which excludes contamination by leukocytes and erythrocytes, is mandatory in this kind of analysis (63, 64). To date, lack of standardization of sample collection and data normalization, along with limited sample size in most of the reports impair the reproducibility and comparability across the studies. Thus, despite the interesting potential of circulating miRNAs as biomarker in HCC, further investigations are still warranted before they can be taken into clinical practice.

##### Prognostic role

microRNAs induce malignant phenotypic changes in liver cancer cells and contribute to the acquisition of invasive and metastatic properties (65, 66). Therefore, it comes with no surprise that their prognostic value has been widely investigated in HCC patients undergoing either curative treatments or local ablative therapies. Despite, Jiang et al. have initially postulated that a global loss of miRNA expression is associated with poorer clinical outcome (67), subsequent analyses in larger cohorts of patients identified single miRNAs as potential prognostic markers. Over-expression of oncogenic miRNAs, such as miR-331-3p (68) or down-regulation of oncosuppressive miRNAs, such as miR-29 (20, 69) has been associated to poor prognosis in unselected cohorts of human HCC tissues. High miR-221 levels were associated with tumor multifocality (15) and reduced time to recurrence after surgery (70). In addition, miR-221 was shown to have a prognostic value, with a significantly lower OS in patients with high-serum miRNA expression (71). Sato et al. looked at miRNA expression in 73 human resected HCCs that had not received any pre-operative therapy and observed that miRNAs expression recapitulates the risk of early or late recurrence in analogy to mRNA profiles (6, 72). The miRNA profile of tumoral tissue could predict early recurrence, while the miRNA profile of non-tumoral tissue was predictive of late recurrence and of *de novo* carcinogenesis. Ji et al. studied miRNA expression in a large (>400 cases) cohort of resected human HCC and observed a remarkable down-regulation of miR-26, which correlated with poor prognosis (19). Patients with high miR-26a expression had longer time to recurrence and longer OS. Despite having worse OS, patients with low miR-26 expressing tumors had an increased benefit from adjuvant therapy with interferon (IFN)-alpha compared to those with tumors with high miR-26a. This was due to the parallel predominant activation of the signaling pathway NF-KB and IL-6 in this cohort of patients, with enhanced oncogenic potential. Thus, miR-26 expression acts not only as a prognostic factor but also as an independent predictor of the response to IFN-alpha (19). On the bases of these findings, a clinical trial is undergoing to look at the effect of adjuvant IFN in patients with HBV-related HCC and low miR-26 expression, and to our knowledge, this is the only clinical trial including miRNA expression as inclusion criteria or stratifying factor, to date. The possibility to detect circulating miRNAs in the sera/plasma of liver cancer patients has recently increased the potential of studying the prognostic values of miRNAs in the clinical setting, resulting in a growing number of reports on the role of circulating miRNAs in predicting relapse after curative treatment. Sugimachi et al. observed that low expression of circulating exosome-related miR-718 was associated to poor histological differentiation, high incidence of tumors beyond the Milan criteria, and a trend to increased recurrence after liver transplantation (73). Some authors have suggested that serum miRNAs levels can be monitored after radical resection to assess disease relapse (74, 75), while others have noticed a correlation between serum levels of miR-200 and response to TACE (76). In addition, some studies have shown that miRNAs can affect survival by modulating invasiveness and metastasis, i.e., miR-135a was found over-expressed in portal vein thrombus tissues and was related to poor clinical outcome (77). Luk et al. (78) found that all miRNAs included in the DLK1–DIO3 cluster at 14q32.2 are coordinately up-regulated in a subset of HCC patients with stem-like features, vascular invasion, and shorter survival. However, the limited sample size of these studies warrants further confirmations before these findings can be incorporated into clinical management.

##### Predictive role

Some miRNAs were found to be predictors of response to anticancer therapy in HCC both *in vitro* and *in vivo*. We have shown that miR-193b can facilitate sorafenib-induced apoptosis through modulation of myeloid leukemia cell differentiation protein (Mcl-1) in human HCC cell lines (79). Similarly, restoration of miR-193b was shown to sensitize HBV+ HCC cells to sorafenib (80). However, expression of this miRNA in patient samples has not been investigated yet. Loss of miR-122 was associated to lower sensitivity of HCC cells to sorafenib in *in vitro* experiments (81). A retrospective analysis showed that high levels of miR-425-3p in HCC bioptic tissues were associated with longer time to progression and OS in patients treated with sorafenib (82). However, further prospective evaluation is needed before miRNAs can be used as stratifying factors for first line treatment. Moreover, given the lack of liver biopsies in the routine clinical setting studies on the role of circulating miRNAs on the prediction of response to sorafenib are likely to represent more useful tools to customize treatment.

##### Therapeutic role

Several approaches to normalize ncRNA expression have been described to date. Therapeutic inhibition of oncogenic miRNAs can be managed through different technologies. Antisense oligonucleotides (ASO) are single-strand DNA molecules that pair to complementary RNA. They may be delivered intravenously but have poor stability (83). On the contrary, locked nucleic acid antimiRs (LNA-antimiRs) seem to be more stable and specific than ASO as they are composed of DNA and a phosphorothioate backbone (84). miRNA sponges contain binding site for several miRNAs and act as competitive inhibitors. They are potentially useful to inhibit a plethora of miRNAs that finally act on the same pathways, or a number of isoforms of the same miRNA (85). The best example of effective anti-miRNA therapy comes from the Miraversen studies. Miravirsen is a LNA-antimiR against miR-122, which is known to promote HCV RNA accumulation within the cells. Miraversen was tested in 36 patients with HCV genotype 1 infection within a phase II clinical trial. Treatment resulted in a dose dependent and prolonged decrease of HCV RNA that lasted beyond the end of active therapy and was not associated with viral resistance and dose-limiting adverse events (86). Given the association noticed between reduced levels of miR-122 and development of liver cancer, the safety of anti-miR-122 therapy has been carefully evaluated, and none of the patients treated with Miravirsen were reported to have developed HCC or other liver-related complications (87).

With regards to HCC treatment, preclinical studies have been successful in achieving tumor growth inhibition through silencing of miR-221/222. MiR-221 and -222 are encoded in tandem from a gene cluster on the chromosome X (Xp11.3) and share the same 5′ region. They regulate and promote cell-cycle progression through down-regulation of cyclin-dependent kinase inhibitors (p27Kip1 and CDKN1C/p57) (88), and pro-apoptotic proteins Bcl-2-modifying factor (Bmf) (15), and can also modulate cell survival through controlling the phosphoinositide 3-kinase–protein kinase B–mammalian target of rapamycin (PI3K–AKT–mTOR) pathway (18). miR-221/222 is frequently up-regulated in human HCC and it was found to be associated with aggressive clinical features. Some reports suggest that *MET* induces miR-221/222 transcription through the activator protein-1 (AP-1) transcription factor, and that miR-221/222 can account for the aggressive biology of the “high *MET*” liver cancers (89). Intravenous delivery of cholesterol- and 2′-*O*-methyl phosphorothioate-modified anti-miR-221 oligonucleotide (anti-miR oligonucleotide, AMO) led to reduced tumor cell proliferation, increased apoptosis, cell-cycle arrest, and increased survival in an orthotopic mouse model of HCC (90). Delivery of AMO anti-miR-221 confirmed anti-tumor effects in a transgenic mouse model over-expressing miR-221 in the liver, which spontaneously develops HCC and accelerates diethylnitrosamine-induced HCC growth (17). Interestingly, three injections over a period of 30 days were sufficient to inhibit miR-221 expression and to cause HCC growth inhibition in this model (17). More recently, Callegari et al. have provided evidence that adeno-associated viruses (AAV) genetically modified to drive the expression of multiple binding sites for miR-221 can act as sponges that sequester miR-221 cellular molecules and exhibit anti-tumor activity in HCC cells and may be tested for *in vivo* miRNA inhibition (91).

miRNA-based therapeutics have also been developed to restore the expression of miRNAs, which are down-regulated in liver cancer. Delivery of miR-26 was attempted trough viral delivery in a mouse model of HCC and proved to be successful in inhibiting cancer cell proliferation, inducing of tumor-specific apoptosis, and blocking disease progression without liver toxicities (92). Intrahepatic delivery of an adenovirus expressing miR-199a in newborn mice led to virus replication and fast removal of implanted HepG2 liver cancer cells, as well as reduced tumor growth in different HCC mouse models (93). Selected evidences showed miR34a is lost in HCC and its expression has been linked to the status of *p53* (94, 95). Thus, attempts to over-express miR-34 have been pursued for the treatment of liver cancer. On one hand, a small molecular modulator termed Rubone, was shown to induce miR34a expression specifically in HCC cells by enhancing the occupancy of *p53* on the miR34a promoter, and showed anti-tumor activity in a xenograft HCC mouse model (96). In *p53* non-deleted HCC, Rubone exhibited a preclinical anti-HCC potency comparable to sorafenib without showing any additional toxicity (96). Therapeutics based on the restoration of miR-34 expression has also been pursued through liposome-mediated miRNA delivery technologies. Indeed, one ongoing phase I study is evaluating the safety of MRX34, a liposomal formulation of miR-34, in patients with primary HCC or those with liver metastases from other cancers (97). This is the first and only example of clinical trial, which assess miRNA-based therapeutics in humans, and results are highly expected from the scientific community. Therapies aiming at blocking or restoring miRNAs are promising and could become a new cornerstone in the treatment of HCC, either in monotherapy or in combination with sorafenib. However, additional investigations are needed to establish the real therapeutic benefit of these approaches, leading eventually to an improvement of survival in HCC.

### Long non-coding RNAs

Long non-coding RNAs can vary in length from 200 nt to 100 kb. Although the majority of lncRNAs have yet to be characterized thoroughly, they have been shown to exhibit cell type-specific expression, localization to subcellular compartments, and association with cancer. lncRNAs can be intronic or intergenic and can be transcribed either in sense or antisense. Their sequence is characterized by a paucity of introns and low-cytosine–guanine (CG) content, which may account for the low level of expression of these transcripts (98). *In vitro* analyses have shown that antisense and intergenic lncRNAs are more stable than others (99). Growing evidence is supporting the involvement of lncRNAs in carcinogenesis. They may modulate cancer initiation and progression by affecting several biological pathways (100). However, their actual mechanism of function is not yet clear. Some evidence suggests that lncRNAs can modulate gene activity and affect the expression of other protein-coding genes. For instance, X-inactive specific transcript (XIST) was shown to modulate gene transcription by “coating Chromosome X” and creating a nuclear compartment that excludes RNA Polymerase-II (RNAPol-II) (101). Interaction with proteins has also been postulated, i.e., lncRNAs interact with the histone modification proteins and act as scaffold molecules for chromatin remodeling complexes (102). Growing evidence is now suggesting that lncRNAs can modulate the microRNome by binding one or multiple miRNAs and act like miRNA sponges (103).

#### Long Non-Coding RNAs in HCC

Long non-coding RNAs have been found to be aberrantly expressed in HCC and to play a role in modulating malignant phenotypes (Table 2). Maternally expressed gene 3 (MEG3) is an imprinted ncRNA located on chromosome 14q32.3 within the *DLK-1* locus. We observed down-regulation of *MEG3* in human HCC tissues in comparison to cirrhotic tissues secondary to hypermethylation of its promoter. *In vitro* experiments confirmed its oncosuppressive properties as over-expression of *MEG3* reduced tumor cell growth and induced apoptosis (104). A recent report confirmed down-regulation of *MEG3* in more than 70 cases of human HCC and found an association between low levels of *MEG3* and poor clinical outcome with reduced overall and relapse-free survival (105). *MEG3* was found to be the most frequently deregulated ncRNA in primary HCC when a set of 16 expression profiles from Oncomine comprising altogether 953 primary human HCC specimens was analyzed (106). Indeed, it was down-regulated in 60% of cases and there was correlation between increased DNA methylation and reduced *MEG3* expression. Interestingly, no alterations in DNA methylation at the *DLK1-MEG3* imprinting locus were found in hepatocellular adenomas or focal nodular hyperplasia. The metastatic lung adenocarcinoma transcript 1 (MALAT-1) is a lncRNA located on chromosome 11q13.1 that was initially identified in metastatic lung cancer and was then found increased in a mouse model of HCC as well as in human HCC cell lines and tissues (107, 108). MALAT-1 seems to mediate carcinogenesis by modulating apoptosis and cell growth in HCC cell lines and its expression correlates with risk of recurrence after liver transplantation (107). HOX transcript antisense RNA (HOTAIR) is expressed from the developmental *HOX-C* locus located on chromosome 12q13.13. HOTAIR expression was found increased in patients with large primary HCC and those with nodal involvement (109, 110). It acts as an oncogene, which may positively regulate the expression levels of multiple genes involved in the promotion of metastatic process, such as vascular endothelial growth factor (VEGF) and matrix metallopeptidase 9 (MMP9) (109). HOTAIR acts as a scaffolding molecule that binds polycomb repressive complex 2 (PRC2) and lysine-specific demethylase 1 (LSD1) and increases recruitment of enzymes involved in the epigenetic modification, with subsequent repression of tumor suppressor genes (102). HOXA transcript at the distal tip (HOTTIP) is another lncRNA located in physical contiguity with a *HOX* locus. HOTTIP is located at the distal tip of the *HOXA13* gene, which encodes transcription factors regulating embryonic development. HOTTIP directly controls *HOXA* locus gene expression, but is also controlled by *HOXA13* showing that a fine regulatory feedback loop is necessary for its activation (111). HOTTIP is remarkably increased in human HCC tissues from liver biopsies and its expression predicts clinical outcome in patients who had not received any HCC treatments, suggesting that liver biopsy may be an important source of information for the understanding of molecular biology of inoperable HCC and for the definition of molecular prognostic markers. Highly up-regulated in liver cancer (HULC) is a <500 nt lncRNA, which show high conservation across species (112). It is present in the cytoplasm of liver cancer cells, where it localizes within the ribosomes, suggesting it may modulate translational activity. Indeed, silencing of HULC in HCC cells-induced global mRNA changes in genes involved in hepatocarcinogenesis (112). Interestingly, not only HULC increased in liver cancer tissues but was also found to be increased in cells from peripheral bloods of HCC patients, suggesting that its involvement in liver cancer may be exerted also through a modulation of the immune system. HULC expression positively correlates with that of HBV x. HBV x was shown to upregulate HULC, which in turn promotes proliferation of hepatoma cells by suppressing the oncosuppressor *p18* (113). In other studies, HULC was shown to promote hepatoma cells proliferation by modulation of lipid metabolism (114). Moreover, a particular variant genotype (rs 7763881) in *HULC* has been found to contribute to decreased HCC development in HBV+ patients (115). Microvascular invasion in HCC (MVIH) is a long ncRNA independently transcribed in human HBV-related HCC tissues compared to adjacent normal counterparts (116). Over-expression of MVIH correlated to microvessel invasion, advanced stage, and poorer OS in a large and unselected cohort of human HBV–HCC patients. Interestingly, MVIH could also significantly predict relapse in patients with early HCC who underwent radical treatment. *In vitro* experiments have shown that MVIH can physically interact with the protein phosphoglycerate kinase 1 (PGK1), an anti-angiogenic protein. It looks like the interaction results in reduced secretion of PGK1, and enhancement of angiogenesis and tumor growth. Indeed, MVIH expression in the primary tumor inversely correlated with PGK1 levels in serum of HCC patients (116). RERT is an lncRNA whose sequence overlaps with that of Prolyl-hydroxylase 1 (*EGLN2*) (117). EGLN2 is one of the three enzymes able to determine degradation of hypoxia inducible factor (HIF) by poly-ubiquitylation and proteasomal degradation. It was shown that a 4-bp deletion polymorphism (rs10680577) within *RERT* significantly correlated with higher expression of RERT and subsequent up-regulation of EGLN2 in human HCC (117). Finally, EGLN2 over-expression made cells more sensitive to hypoxia stress, leading to less HIF-alpha stabilization and HIF activation, which were detrimental for hepatic cell survival. These findings provided an example of how up-regulation of lncRNA can promote hepatocarcinogenesis through regulation of transcription of close genes and modulation of cell response to stress (117). Long intergenic ncRNA regulator of reprograming (lincRNA-RoR) is another hypoxia-responsive lncRNA, which is increased in malignant human liver cancer cells, and in the hypoxic regions of tumor cell xenografts *in vivo* (118). Interestingly, linc-ROR was detected in extracellular vesicles released by tumor cells during hypoxia, suggesting that this lncRNA may contribute to the intercellular signaling promoting cell survival in hypoxic stress (118). High expression in HCC (HEIH) is an lncRNA identified in human liver tissues and was named after its over-expression in human HBV–HCC compared to cirrhotic samples (119). It was shown to act as an oncogene *in vitro* and *in vivo* models and was found to interact with the enhancer of zeste homolog 2, an essential subunit of PRC2 complex. HEIH over-expression was found significantly associated with higher recurrence in HBV–HCC patients and was an independent prognostic factor for OS (119). lincRNA–UFC1 was found over-expressed in HCC tissues and associated with advanced stages and poor clinical outcome (120). lincRNA–UFC1 seems to control expression of beta-catenin not by direct interaction but through the binding to HuR, a RNA-binding proteins that can in turn interact with beta-catenin mRNA. Interestingly, lincRNA–UFC1 was also found to be a direct target of miR-34, whose loss in HCC was postulated to be the driver of lincRNA–UFC1 over-expression (120).

**Table 2:** **Long non-coding RNAs involved in HCC pathogenesis and development**.

lncRNA	Expression in HCC compared to normal liver	Cell phenotype	Source	Reference
MEG3	Down	Growth, reduced apoptosis	Human tissues and cell lines	(104–106)
			Mouse tissues	
MALAT-1	Up	Growth, reduced apoptosis	Human tissues, cell lines, plasma and sera	(107, 108, 132, 137)
			Mouse tissues	
HOTAIR	Up	Progression, migration, invasion	Human tissues	(109, 110)
HOTTIP	Up	Progression, invasion	Human tissues and cell lines	(111)
HULC	Up	Proliferation	Human tissues and cell lines	(112, 134)
MVIH	Up	Invasion, migration	Human tissues and cell lines	(116)
RERT	Up	Hepatocarcinogenesis	Human tissues and cell lines	(117)
LincRNA-RoR	Up	Survival	Human cell lines	(118)
			Mouse tissues	
HEIH	Up	Cell-cycle progression	Human tissues	(119)
TUC338	Up	Cell-cycle progression, cell growth	Human tissues and cell lines	(125)
			Mouse cell lines	
H19	Up	Cells differentiation	Human tissues	(135)
			Mouse tissues and cell lines	

#### Transcribed Ultra-Conserved Regions

Ultra-conserved regions (*UCRs*) comprise 481 genomic sequences longer than 200 bp, which are totally conserved among mouse, rat, and human genomes (121). SNPs and mutations are normally under-represented in *UCR* genes (122). Many *UCR*s are transcribed (T-UCRs) in normal human tissue (123). Some of them have a ubiquitous expression, while others are tissue-specific (124). T-UCRs were shown to have distinct genome-wide expression profiles in different human cancers and this evidence supports their role in human carcinogenesis (123–127). The function of T-UCRs is still partly unknown, but they seem to modify the microRNome of the cell (128). Several mechanisms may be responsible for the deregulation of T-UCRs, including promoter methylation (127) and transcriptional activation (123). T-UCRs have been investigated in HCC and their expression profile was found to be deregulated in malignant compared to normal hepatocytes (125). The ultra-conserved element 338 (*uc.338*) is partly overlapping a protein-coding gene but was found to be transcribed as part of an independent lncRNA (TUC338). TUC338 expression is increased in human and murine malignant hepatocytes as well as in human HCC tissues in comparison to normal liver. Interestingly, not only the sequence but also the functional activity in promoting cellular growth is conserved across the species, suggesting that TUC338 is essential for the normal homeostasis of liver cells and that its aberrant over-expression may be responsible for driving carcinogenesis (125). Kogure et al. have also demonstrated that T-UCRs can be found in extracellular vesicles secreted by cancer cells, strengthening the hypothesis that they exert an important role in modulating tumor cell growth and they are part of the intercellular signaling through which HCC may grow and spread (129). Growing evidence is supporting the role of T-UCRs also in other types of cancer (124, 126, 130), and given their conservation it is likely that they play an essential part in driving carcinogenesis and may therefore represent valuable targets for novel therapeutics.

### Interactions between lncRNAs and miRNAs

Recent evidence suggests that miRNAs and lncRNAs are likely to modulate each other by acting in a complex network. miRNAs can directly or indirectly regulate the expression of lncRNAs. Calin et al. showed that miRNAs can bind to the sequence of lncRNAs and negatively regulate their expression (123). MALAT-1 was found to be a target of miR-125b, which can directly bind the lncRNA and control its expression in bladder cancer (131). Over-expression of MALAT-1 (107) and down-regulation of miR-125 (132) have been separately reported in human HCC, suggesting that this mechanism may account for up-regulation of MALAT-1 in liver cancer as well. miRNAs were shown to control lncRNA expression also through indirect mechanisms. For instance, the miRNA-dependent control of the methylation machinery may have implications on the oncogenic role of selected lncRNAs in HCC. This is the case of MEG3 and miR-29. MiR-29 can modulate *de novo* methyltranferase (DMNT) 1 and 3. In case of low expression of miR-29, methylation-dependent tissue-specific regulation of MEG3 does not occur, and the lncRNA is suppressed (104).

If miRNAs can regulate lncRNAs, there is also evidence that lncRNAs may, in turn, regulate miRNAs by acting as competing endogenous RNAs or by being processed into small RNAs (133). HULC was shown to have binding sites for miR-372 and therefore to act as an endogenous “sponge.” HULC-mediated inhibition of miR-372 leads to reduction in repression of the target gene *PRKACB* and subsequent phosphorylation of the proto-oncogene c-AMP response element-binding protein (CREB) in HCC cells (134). Linc-RoR, a hypoxia-induced lncRNA, was shown to deplete HCC cells from miR-145 in hypoxic conditions and to mediate its biological effect through the interaction with miRNAs (118). Liz et al. have recently provided solid evidence that T-UCR can affect miRNA processing by preventing the release of mature forms. Indeed, uc.283 was shown to interact with the lower stem region of pri-miR-195 transcript preventing the miRNA cleavage by Drosha (128). Some authors have reported that the lncRNA H19, observed to be up-regulated in HCC (135), can mediate muscle differentiation by releasing miR-675, which is encoded within its sequence (136). Ren et al. have speculated that MALAT-1 can be fragmented in several small RNAs that can then be released from cancer cells and found in the plasma of HCC patients (137). Despite growing evidence is reported on the interplay between different classes of ncRNAs, further investigation is warranted in order to better understand the function of ncRNA and exploit their potential as therapeutic targets.

## Conclusion

Non-coding RNAs participate in genomic regulation from transcription, post-transcription, and epigenetic modification. They can interact with each other and create networks of signal transduction that have a crucial role in hepatocarcinogenesis and disease progression. Moreover, they may modulate cell response to anticancer agents and have an intrinsic antineoplastic effect complementary to that of systemic therapies. In recent years, a large number of ncRNAs have been identified and several studies have investigated the role of ncRNA in HCC providing valuable knowledge for the understanding of liver carcinogenesis. However, despite the increasing knowledge on ncRNAs in liver cancer, none of them has entered clinical practice, and only few phase I and II trials have been conducted up to now. The process of translation of preclinical results into clinic seems challenging and hard to pursue. Indeed, the safety of ncRNAs as clinical therapeutic targets needs to be established with certainty. For example, the use of viral delivery systems may activate the innate immune responses with subsequent serious adverse events, while targeting ncRNAs involved in regulation of gene expression could bring to unexpected off target gene effects. Moreover, modulation of miRNA expression and competition with endogenous miRNAs may have unexpected effects on cellular physiology.

Hepatocellular carcinoma has still poor prognosis and limited therapeutic options. To date, only one drug, sorafenib, has been approved for first line treatment, and several trials investigating novel drugs have failed. Several ncRNAs directly involved in HCC promotion and progression have been effectively targeted in preclinical studies. It is hoped that these efforts will be soon translated into clinical practice.

## Conflict of Interest Statement

The authors declare that the research was conducted in the absence of any commercial or financial relationships that could be construed as a potential conflict of interest.
